# Membranous urethral length measurement on preoperative MRI to predict incontinence after radical prostatectomy: a literature review towards a proposal for measurement standardization

**DOI:** 10.1007/s00330-023-10180-7

**Published:** 2023-09-22

**Authors:** Thierry N. Boellaard, Margriet C. van Dijk-de Haan, Stijn W. T. P. J. Heijmink, Corinne N. Tillier, Hans Veerman, Laura S. Mertens, Henk G. van der Poel, Pim J. van Leeuwen, Ivo G. Schoots

**Affiliations:** 1 Department of Radiology, Netherlands Cancer Institute, Plesmanlaan 121, 1066 CX Amsterdam, the Netherlands; 2 Department of Urology, Netherlands Cancer Institute, Amsterdam, the Netherlands; 3 Department of Urology, Amsterdam University Medical Centers, Amsterdam, the Netherlands; 4 Department of Radiology and Nuclear Medicine, Erasmus University Medical Center, Rotterdam, the Netherlands

**Keywords:** Urethra, Prostatectomy, Urinary incontinence, Prostatic neoplasms, Magnetic resonance imaging

## Abstract

**Objectives:**

To investigate the membranous urethral length (MUL) measurement and its interobserver agreement, and propose literature-based recommendations to standardize MUL measurement for increasing interobserver agreement. MUL measurements based on prostate MRI scans, for urinary incontinence risk assessment before radical prostatectomy (RP), may influence treatment decision-making in men with localised prostate cancer. Before implementation in clinical practise, MRI-based MUL measurements need standardization to improve observer agreement.

**Methods:**

Online libraries were searched up to August 5, 2022, on MUL measurements. Two reviewers performed article selection and critical appraisal. Papers reporting on preoperative MUL measurements and urinary continence correlation were selected. Extracted information included measuring procedures, MRI sequences, population mean/median values, and observer agreement.

**Results:**

Fifty papers were included. Studies that specified the MRI sequence used T2-weighted images and used either coronal images (*n* = 13), sagittal images (*n* = 18), or both (*n* = 12) for MUL measurements. ‘Prostatic apex’ was the most common description of the proximal membranous urethra landmark and ‘level/entry of the urethra into the penile bulb’ was the most common description of the distal landmark. Population mean (median) MUL value range was 10.4–17.1 mm (7.3–17.3 mm), suggesting either population or measurement differences. Detailed measurement technique descriptions for reproducibility were lacking. Recommendations on MRI-based MUL measurement were formulated by using anatomical landmarks and detailed descriptions and illustrations.

**Conclusions:**

In order to improve on measurement variability, a literature-based measuring method of the MUL was proposed, supported by several illustrative case studies, in an attempt to standardize MRI-based MUL measurements for appropriate urinary incontinence risk preoperatively.

**Clinical relevance statement:**

Implementation of MUL measurements into clinical practise for personalized post-prostatectomy continence prediction is hampered by lack of standardization and suboptimal interobserver agreement. Our proposed standardized MUL measurement aims to facilitate standardization and to improve the interobserver agreement.

**Key Points:**

• *Variable approaches for membranous urethral length measurement are being used, without detailed description and with substantial differences in length of the membranous urethra, hampering standardization*.

• *Limited interobserver agreement for membranous urethral length measurement was observed in several studies, while preoperative incontinence risk assessment necessitates high interobserver agreement*.

• *Literature-based recommendations are proposed to standardize MRI-based membranous urethral length measurement for increasing interobserver agreement and improving preoperative incontinence risk assessment, using anatomical landmarks on sagittal T2-weighted images*.

**Supplementary information:**

The online version contains supplementary material available at 10.1007/s00330-023-10180-7.

## Introduction

In men with localized prostate cancer, several (curative) treatment options are available, such as radical prostatectomy (RP), external beam radiotherapy, brachytherapy, and active surveillance, all with good oncological outcome [1]. The oncological benefit of each treatment should be carefully weighed against the risk in terms of side effects by both the physician and patient (shared decision-making). The major potential side effects of RP are urinary incontinence and erectile dysfunction, both impacting on quality of life [2]. Counselling patients about these potential side effects is part of the shared decision-making on treatment [1]. Algorithms on individual risk assessment on postoperative urinary incontinence are available, guiding this counselling process [3].

Besides patient-related factors (e.g. age, pre-existing lower urinary tract symptoms (LUTS), and body mass index (BMI)) and surgical factors (e.g. nerve sparing), it was reported that magnetic resonance imaging (MRI)–based anatomical related factors may improve the individual risk assessment of incontinence after RP [4, 5]. The most studied MRI parameter has been the membranous urethral length (MUL). Recent meta-analyses have shown the predictive value of the MRI-based MUL measurement [6, 7] with larger MUL is associated with significantly greater odds for return to continence [7].

The potential impact of pre-treatment incontinence risk assessment for treatment decision-making including the MUL as input parameter is embraced in urological surgical practices [3]. Several institutions have adopted their own prediction models and have calculated their own threshold for low- and high-risk postoperative (in)continence, including the MUL [3, 8, 9]. However, before implementation into broad clinical practices, there should be agreement on the standardized approach of MUL measurement.

The purpose of this review was to investigate the current literature on the utility of MUL measurement, to identify objective findings regarding MRI acquisition, anatomical landmarks, and measurement definitions, and to propose the first literature-based recommendations on how MUL measurement on pre-treatment MRI scans should be performed.

## Methods

### Objective

We investigated the literature on published MUL measurements, including measuring approaches, MRI sequences used, population mean/median values, type of observer, and observer agreement. We proposed recommendations to standardize MRI-based MUL measurement using anatomical landmarks, with detailed descriptions and illustrations of MUL measurements and measurement pitfalls.

### Search strategy

A systematic search was conducted using the Embase, Medline ALL Ovid, Web of Science Core Collection, Cochrane CENTRAL register of trials, and Google Scholar databases up to August 5, 2022, without restrictions regarding publication date or language (supplementary material, appendix 1). The literature search was conducted by a medical librarian. References from selected studies were also screened. This search was also used in a previous publication, but has been updated [6].

### Inclusion criteria

The study population was limited to men with non-metastasized primary diagnose prostate cancer who underwent RP using any route or approach. Randomized controlled trials and prospective and retrospective cohort studies reporting data on preoperative MRI-based MUL measurements and follow-up data on urinary continence were included. There were no restrictions on follow-up time. We excluded unpublished data, conference abstracts, and review articles. We also excluded studies with the smallest number of patients for published papers using the same data sets (in case of complete overlapping data).

### Data extraction

We followed the Preferred Reporting Items for Systematic Reviews and Meta-analyses (PRISMA) process for reporting study inclusion and exclusion [10]. The abstract and full-text screening and subsequent data extraction were carried out by two researchers independently (M.C.d.H. and T.N.B.). Discrepancies between the reviewers were resolved via discussion (M.C.d.H., T.N.B., and I.G.S.). A data extraction form was developed to collect information on the patient characteristics and study methodology (surgical technique, MRI protocol, questionnaires, and continence follow-up protocols). More detailed data extraction on MUL measurement methodology used (MRI sequence, image orientation, landmarks, agreement) was performed by one researcher (T.N.B.).

### Statistical analysis

This literature review refers to descriptive data; therefore, statistical analysis was not performed.

## Results

### Study parameters

We included 50 papers (Table 1), widely distributed over the world, dominated by South Korea (*n* = 16), the USA (*n* = 10), and Japan (*n* = 9). The studies cover 18,545 men with pre-treatment MRI.

**Table 1:** Study parameters, MRI sequences, anatomical landmarks and lengths

Author, publication year	Subjects (*n* =)	MUL (mm)	Follow-up moments continence post-prostatectomy	Prostate size	Definition of MUL measurement (description in methods)	MRI sequence	Orientation	Proximal MU landmark	Distal MU landmark	Reader	Country
Cho, 2015 [17]	27	11.3 ± 1.6*	1, 3, 6, 9, and 12 months	39.4 ± 14.2 g	A straight line between the prostatic apex and the penile bulb was drawn in the mid-sagittal plane and the coronal plane	T2 TSE	Sagittal and coronal (mean)	Prostatic apex	Penile bulb	Radiologist	South Korea
Choi, 2015 [18]	158	11.8/11.9*	1, 3, and 6 months	31/33.2 ml	The length from the prostatic apex to the level of the urethra at the penile bulb in the midline sagittal plane	T2	Sagittal	Prostatic apex	Level of the urethra at the penile bulb	NA	South Korea
Coakley, 2002 [19]	180	14 (6–24)*	12 months	NA	The distance from the prostatic apex to the entry of the urethra into the penile bulb	T2 FSE	Coronal	Prostatic apex	Entry of the urethra into the penile bulb	1 of 2 readers	USA
Fukui, 2019 [20]	270	7.3**#**	1, 3, 6, and 12 months	NA	NA	T2^i^	Sagittal	NA	NA	NA	Japan
Greenberg 2022 [21]	251	14 (12–17)# rad 15 (12–18)# uro	Within 6, 12, and 24 months	34 (27–44) cc	From the lowest of the prostatic apex to the entry of the urethra into the penile bulb	T2^i^	Sagittal (uro)	Prostatic apex	Entry of the urethra into the penile bulb	22 radiologists 1 urologist	USA
Grivas, 2018 [8]	439	12.9 (1.7)/11.5 (1.5)*	6, 12, 18, and 24 months	49.2 cm^3^ (68.1)/44.5 cm^3^ (19.3)	NA	T2 TSE^a^	Coronal and sagittal	NA	NA	1 urologist 1 radiologist	The Netherlands
Grivas, 2017 [22]	49	16.2 (14.1–18.4)**#**	6 and 12 months	62 cm^3^ (55–78)	NA	T2 TSE^a^	Sagittal^a^	NA	NA	1 urologist 1 radiologist	The Netherlands
Hakimi, 2011 [23]	75	14.6 (8–26)*	1.5, 3, 6, 9, 12, 15, and 18 months	58.4 (28–185) g	The distance from the apex of the prostate to the bulb	T2	Coronal^i^	Apex of the prostate	Bulb	1 of 2 radiologists	USA
Hikita, 2020 [24]	119	12.1 (8.9–16.1)#	1, 3, 6, 9, and 12 months	26.0 (9.6–66.1) ml	The distance from the prostatic apex to the level of the urethra at the penile bulb	T2	Coronal	Prostatic apex	Level of the urethra at the penile bulb	NA	Japan
Hoeh 2022 [25]	68	14.7 (13.0–16.7)# cor 15.1 (12.8, 16.8)# sag	> 6 months	35 (28–45) ml	NA	T2^i^	Sagittal and coronal	NA	NA	Specialist in urologic imaging, supervised by a board-certificated radiologist	Germany
Hong, 2009 [26]	141	NA	6 months	38.4 (17–88) g	NA	NA	NA	NA	NA	2 radiologists	South Korea
Iacovelli 2022 [27]	100	NA^s^	1, 3, 6, and 12 months	NA^s^	NA	NA	Sagittal	NA	NA	Two radiologists	Italy
Ikarashi, 2018 [28]	204 (10 were incontinent preoperative)	13.1 (4.5–22.9)#	3 (at least), 6, 9, and (max) 12 months	38 (7–94) g	A distance from the prostatic apex to the level of the urethra at the penile bulb	T2	Coronal and sagittal	Prostatic apex	Level of the urethra at the penile bulb	Several urologists and a researcher	Japan
Jeong, 2012 [29]	708	NA^s^	3, 6, 9, 12, 15, 18, 21, and 24 months	NA	NA	NA	NA	NA	NA	NA	South Korea
Jeong, 2013 [30]	731	12.8 (6–23)*	2 weeks, 1, 3, 6, 9, 12, and 24 months	38.5 (16.0–141.0) ml	The distance from the prostatic apex to the entry of the urethra into the penile bulb	T2	Coronal	Prostatic apex	Entry of the urethra into the penile bulb	2 uro-radiologists	South Korea
Jeong, 2014 [31]	1168	NA^s^	1, 3, and 12 months	NA	NA	NA	NA	NA	NA	NA	South Korea
Kadono, 2016 [32]	111	13.6 ± 2.4*	12 months	40.4 ± 9.0 ml	The distance from prostatic apex to the entry of the urethra into the penile bulb	NA	Coronal	Prostatic apex	Entry of the urethra into the penile bulb	NA	Japan
Kim, 2011 [33]	763 (3.9% preoperative incontinent)	NA^s^	1, 3, 6, 9, 12, 18, and 24 months	NA	From prostatic apex to the level of the urethra at penile bulb	NA	Sagittal	Prostatic apex	Level of the urethra at penile bulb	NA	South Korea
Kim, 2020 [34]	190	14.6 ± 3.0 cor/14.2 ± 2.7 sag*	8 weeks, 3, 6, 9, and 12 months	36.4 (29.5–45) cm^3^	From the inferior end of the prostatic apex to the level of the penile bulb	T2^i^	Coronal and sagittal	Inferior end of the prostatic apex	Level of the penile bulb	1 uroradiologist 1 uro-oncology fellow 1 urologist	UK
Kim, 2019 [35]	529	12.3 ± 4.5* 11.7**#**	1, 3, 6, and 12 months	35.6 ± 14.6, 32.0	The distance from the posterior prostate apex to the urethra level at the penile bulb	T2^i^	Sagittal	Posterior prostate apex	Urethra level at the penile bulb	NA	South Korea
Kitamura, 2019 [36]	320	10.5 (9.3–11.5)#	1, 3, 6, and 12 months	25.0 (19.0–35.0)	The distance from the prostatic apex to the level of the urethra at the penile bulb	T2	Sagittal	Prostatic apex	Level of the urethra at the penile bulb	1 urologist	Japan
Ko, 2020 [37]	123	NA	1–3 months	NA	The distance from the prostatic apex to the entry of the urethra into the penile bulb	NA	Coronal and sagittal	Prostatic apex	Entry of the urethra into the penile bulb	1 radiologist	USA
Kohjimoto, 2020 [38]	179	17.3 (14.6–19.7)#	3, 6, 12, and 24 months	NA	The distance from prostatic apex to the entry of urethra into penile bulb	T2	Coronal	Prostatic apex	Entry of urethra into penile bulb	NA	Japan
Lamberg, 2022 [39]	589	NA^s^	3, 6, 12, and 24 months	NA^s^	The distance from the prostate apex to the urethral entry into the penile bulb	T2	Sagittal and coronal	Prostate apex	Penile bulb	Three abdominal radiologists One abdominal radiology fellow	USA
Lee, 2013 [40]	249: 92 early recovery (< 3 months), 157 late recovery (remaining)	NA^s^	Monthly, at least 3 months	NA	The distance from the prostatic apex to the entry of the urethra into the penile bulb	T2	Coronal	Prostatic apex	Entry of the urethra into the penile bulb	NA	South Korea
Lee, 2006 [15]	156	NA	Within 3 months	NA	NA (only reference)	T2 FSE	NA	NA	NA	2 radiologists	South Korea
Lee, 2014 [16]	1011	NA	1, 3, 6, and 12 months	NA	NA (only reference)	NA	Coronal	NA	NA	2 radiologists	South Korea
Lee, 2020 [41]	2310 (of which 610 aged > 70 years)	NA^s^	3 and 12 months	NA	NA	NA	NA	NA	NA	NA	South Korea
Li, 2020 [42]	156	NA	6, 9, and 12 months	31.0 (23.6–40.3)/ 33.8 (22.1–44.8)	The inferior edge of the prostate apex to the superior margin of the penile bulb	T2 FSE	Sagittal^i^	Prostate apex	Superior margin of the penile bulb	NA	China
Lim, 2012 [43]	94	10.4 ± 3.8*	12 months	29.7 ± 13.5 ml	The most prominent portion of the prostate apex to the level of the urethra at the penile bulb	T2	Sagittal (cross-referenced to coronal)	Most prominent portion of the prostate apex	Level of the urethra at the penile bulb	1 radiologist	South Korea
Lin, 2020 [44]	602	14.6 ± 3.6*	3, 6, and 12 months	42.6 ± 21.2	A straight line between the prostatic apex and the penile bulb	NA	Coronal and sagittal	Prostatic apex	Penile bulb	2 readers	Australia
Matsushita, 2015 [45]	2849: 1899 training, 950 validation	12#	At least 6 months, 1 year	31 cm^3^	NA (only reference)	T2 FSE	NA	NA	NA	1 reader (accuracy confirmed by radiologist)	USA?
Mendoza, 2011 [46]	80	17.1 ± 4.5*	Monthly until 6 months	34.7 ± 17.8 g	From the prostate base to the bulb	NA	Coronal	NA	NA	Radiologists	USA
Nguyen, 2008 [47]	274	14.0*	12 months	37.5 (12.6–175.8) cc	The distance from the prostatic apex to the entry of the urethra into the penile bulb. Urethral sphincter anatomy was studied using 3-cross referenced planes of T2-weighted images, that is on the midline sagittal plane with coronal reference and on the coronal plane with axial reference	T2 FSE	Coronal and sagittal, (unclear how combined)	Prostatic apex	Entry of the urethra into the penile bulb	1 reader	USA
Onishi, 2018 [48]	215	12.7 ± 3*	Monthly until patients were continent	40.5 ± 13.8 g	A distance from apex of prostate to the urethra at the level of the penile bulb	T2	Coronal and sagittal (unclear how combined)	Apex of prostate	Urethra at the level of the penile bulb	2 urologists	Japan
Ota, 2021 [49]	50	12.7 (11.2–13.9)/11.3 (10.2–12.8)#	1, 3, 6, and 12 months	27.2 (22.9–37.2) ml/ 22.7 (18.2–29.5) ml	NA	NA	NA	NA	NA	NA	Japan
Oza, 2022 [50]	NA^s^	NA^s^	12 months	33.50 ml	Distance between the apex of the prostate to the bulb of the penis	T2	Sagittal and coronal	Apex of the prostate	Bulb of the penis	Urologist and uro-radiologist	UK
Paparel, 2009 [51]	64 with pre and post OK MRI	14**#**	Median 7 months	NA	The distance from the prostatic apex to the level of the urethra at the penile bulb	T2 FSE	Sagittal (cross-referenced to coronal)	Prostatic apex	Level of urethra at the penile bulb	1 radiologist 1 urologist (consensus)	USA
Park, 2021 [9]	166	14.7 (5.1–24.8)*	3 months	44 (19–150) mm^3^	NA	T2	Sagittal^i^	NA	NA	1 radiologist	South Korea
Regis, 2019 [52]	72	NA	1, 6, and 12 months	41 (15–155) cm^3^	NA	T2	Coronal	NA	NA	1 urologist (in consensus with radiologist if doubtful)	Spain
Sadahira, 2019 [53]	70	? (8.7–14.9)#	12 months	? (8.9–103.7) ml	The entry of the urethra into the penile bulb to the prostatic apex	T2 TSE	Sagittal	Prostatic apex	Entry of the urethra into the penile bulb	1 urologist	Japan
Sauer, 2019 [54]	316	10.5 (5.0–25.0)*	6 and 12 months (and 1 week after catheter removal)	47 (20–160) ml	The distance of two horizontals: (1) on the level of the deepest part of the prostate’s apex and (2) on the highest visible part of the penile bulbous	T2	Sagittal	Deepest part of prostate apex	Highest visible part of the penile bulbous (between 2 horizontal)	2 radiologists	Germany
Schmid, 2020 [55]	42	NA	6 weeks, 3, 6, and 12 months	NA	The distal prostate apex to the proximal penile bulb in sagittal orientation	T2	Sagittal	Distal prostate apex	Proximal penile bulb	NA	Switzerland
Son, 2013 [56]	258 (PALP vs RRP)	13.1 ± 2.4*	2 weeks, 1, 3, 6, 9, and 12 months	43.3 ± 24.9 ml	The distance from the prostatic apex to the entry of the urethra into the penile bulb	T2	Coronal	Prostatic apex	Entry of the urethra into the penile bulb	NA	South Korea
Song, 2017 [57]	186	15.6 ± 2.7* 15.9 (7.2–22.9)**#**	1, 3, 6, and 12 months	34.0 ± 15.9, 30.0 (8.0–113.0) ml	A distance from the apex of prostate to the urethra at the level of the penile bulb	T2	Sagittal (cross-referenced to the coronal)	Apex of prostate	Urethra at the level of the penile bulb	2 non-radiologists	South Korea
Tienza, 2018 [58]	746	NA	12 months	NA	NA	T2	Sagittal	NA	NA	NA	Spain
Tutolo 2022 [59]	209	14 (11–16)#	3 months, 3–6 months, 6–12, and from 12 to last‐month follow‐up	37 (27–51) cm^3^	Distance between the prostate apex and the penile bulb	T2	Sagittal	Prostate apex	Penile bulb	Two urologists (after training by radiologist)	Belgium Italy
Von Bodman, 2012 [60]	600	13 (11–16)#	6 and 12 months	15.8 (12.4–20.0) cm^3^	From the apex of the prostate to the base of the urethral bulbus	T2	Coronal	Apex of the prostate	Base of the urethral bulbus	2 raters trained by a radiologist	USA?
Wenzel, 2021 [61]	128	15 (12–17)#	24 h after catheter removal (routinely between 5 and 7 days after surgery)	40 (30–50)	NA	T2^i^	Sagittal and coronal	NA	NA	Researcher with special training supervised by a radiologist	Germany
Yang, 2020 [62]	150	13.8 ± 3.7*	3, 6, and 12 months	40.1 ± 26.1 ml	A distance from the apex of prostate to the urethra at the level of the penile bulbi	T2	Coronal	Apex of prostate	Urethra at the level of the penile bulbi	NA	China

*FSE* fast spin echo, *MU* membranous urethra, *NA* not available, *TSE* turbo spin echo. *Mean, #median, *a* average, ^i^based on figure in manuscript, ^a^requested from authors, ^s^provided for subgroups only, *rad* radiologist, *uro* urologist

### MRI sequences, anatomical landmarks, and lengths

T2-weighted images for MUL measurement were used in all studies that specified the MRI sequence; either using sagittal images (*n* = 18), coronal images (*n* = 13), or both (*n* = 12) (Table 1).

The anatomical landmark of the proximal end of the membranous urethra (MU) was most commonly described as ‘prostatic apex’.

The anatomical landmark of the distal end of the MU was most commonly described as ‘level of the urethra at the penile bulb’ and ‘entry of the urethra into the penile bulb’. Detailed reproducible measurement technique descriptions were lacking in all studies.

The mean MUL was reported between 10.4 and 17.1 mm and median MUL between 7.3 and 17.3 mm, showing large variations.

Measurements were performed by urologists, radiologists, and trainees.

Articles did not specify the location of the measurement line on sagittal images (e.g. anterior, central, posterior to the urethra) and exact line orientation. In the provided figures in the articles, the location of the measurement line is variable. Additionally, there is no evidence on how to deal with an anterior membranous urethra (MU) overlapping apex.

### Interobserver agreement

Six studies reported on the interobserver agreement. The intraclass correlation coefficient (ICC) was reported by 5 studies, ranging from 0.34 to 0.89 (Table 2).

**Table 2:** Interobserver agreement

Author, publication year	Statistics	Interobserver	Interobserver with training	Intraobserver
Greenberg, 2022 [21]	ICC	0.34 (sag)		
Kim, 2020 [34]	ICC	0.89 (cor) 0.77 (sag)		
Lamberg, 2022 [39]	ICC	0.38 (sag and cor)	0.62 (sag and cor)	
Sauer, 2019 [54]	ICC	0.82 (sag)		
Von Bodman, 2012 [60]	Weighted kappa	0.48 (cor)		
Veerman, 2022 [11] (additional data)	ICC	0.84 (sag)		0.93–0.98

*Cor* coronal, *Sag* sagittal, *ICC* intraclass correlation coefficient

### Recommendations based on literature for reproducible MUL measurement

Based on current observations, we suggest to measure the MUL in a way with high interobserver observer agreement [11]. We propose the following recommendations:

- Acquire high-resolution T2-weighted images, according to PI-RADS guidelines [12], preferably on 3-Tesla scanners, in both sagittal and coronal planes.
- Measure the MUL in sagittal T2-weighted images since the coronal images are usually not angulated parallel to the MU.
- Standardize the measurement approach into the following
  - Identify the hyperintense urethral lumen of the MU on one of the midsagittal images, and the dorsal hypointense membranous structure.
  - Place the measurement line just dorsally from and perpendicular to this hyperintense urethral lumen, from the prostate apex to the penile bulb.
  - Identify the upper (cranial) limit, where the measurement line intersects with the prostate apex defined as the lowest border of the peripheral zone at the dorsal prostate. Scroll parasagittally to the left and right to confirm the lowest border of the peripheral zone. When in doubt, crosslink with coronal images.
  - Identify the lower (caudal) limit, where the MU enters the penile bulb. The landmark for the penile bulb is the intersection of the urethra with the bulb of the corpus spongiosum. Scroll parasagittally to left and right to confirm the border of the penile bulb. When in doubt, crosslink with the coronal images.

### Illustrations of proposed measurement technique

**Fig. 1:**
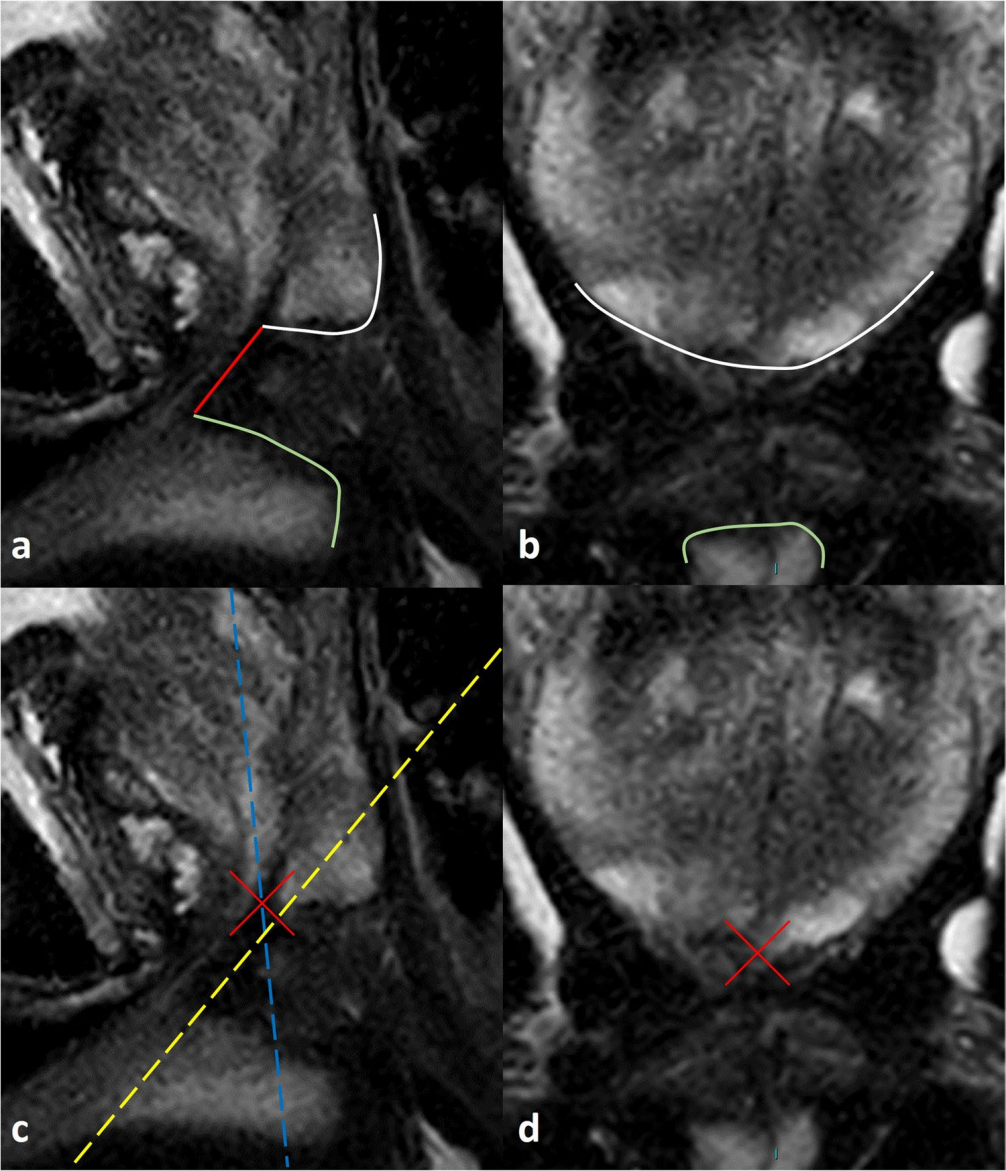
Proposal of membranous urethral length (MUL) measurement on midsagittal MR images. **a** Midsagittal T2w image of the prostate. Sagittal MR images are mandatory for appropriate MUL measurements. The proposed MUL measurement (red line) is determined at the dorsal side of the urethra lumen (this MUL was measured 16 mm). The upper border of the MU is determined by the presence of prostatic tissue. Intraprostatic urethra is excluded from the measurement as intraoperatively sparing is not always performed or possible (i.e. apical tumors). The upper border of the MU is determined by the intersection of the urethra with the dorsally located peripheral zone (white line). The lower border of the MU is determined by the intersection of the urethra with the entrance to penile bulb (green line). **b** Crosslinking of coronal T2w images with sagittal images may help identifying and determining the borders of the anatomical structures related to the MU. Delineation of the peripheral zone (white line) on coronal images informs on the intersection with the MU. **c**, **d** Crosslinking of sagittal (**c**) and coronal (**d**) T2w images with a cross mark (red X) in PACS viewing software may confirm the correct identification of the lower border of the peripheral zone. The scan direction of the coronal images is illustrated by the blue dashed line. Notice that this coronal scan orientation may not be similar to the MU direction (dashed yellow line), which may lead to an inappropriate MUL measurement when coronal images would have been used

**Fig. 2:**
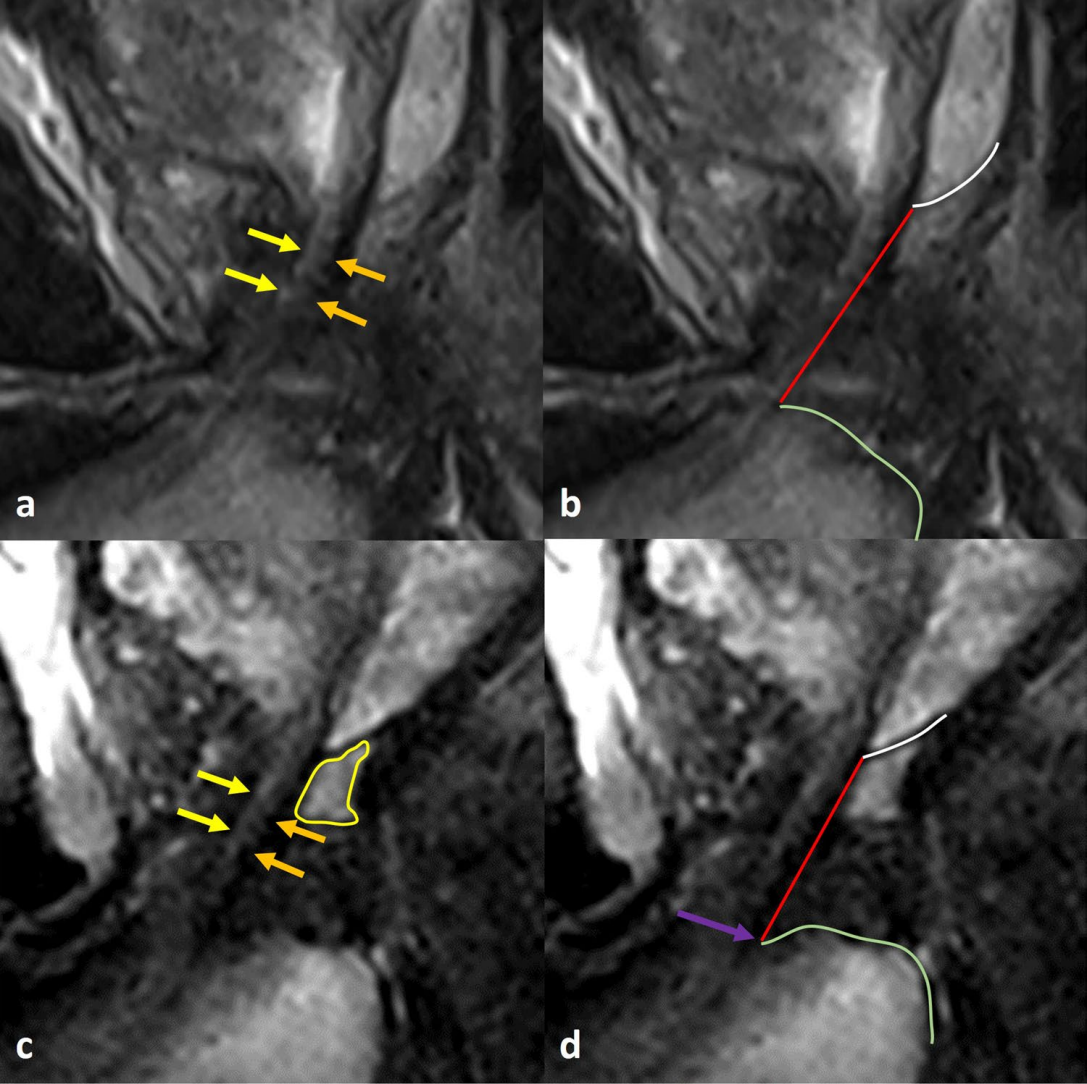
Critical steps in membranous urethral length (MUL) measurement on midsagittal MR images. **a**, **b** A prostate cancer patient with sagittal T2w images on preoperative MRI. In MUL measurement, critical steps need to be distinguished: (1) the hyperintense lumen (yellow arrows) and the hypointense dorsal part (orange arrows) of the membranous urethra need to be identified. (2) The line of the membranous urethra measurement should be placed dorsally and parallel (red line, MUL). (3) The upper border of the measured membranous urethra intersects with the prostate apex, defined as the lower border of the peripheral zone at the dorsal side (white line). (4) The lower border the measured membranous urethra intersects with the penile bulb, the bulb of the corpus spongiosum (green line). **c**, **d** Another prostate cancer patient with sagittal T2w images on preoperative MRI. Notice the difference between hyperintense signal of the peripheral zone and retroprostatic part of the rectovesical space (yellow delineation). Also notice base of the penile bulb can be slightly curved (purple arrow), extending the MUL slightly. The measurement (red line) was 20 mm in this case

**Fig. 3:**
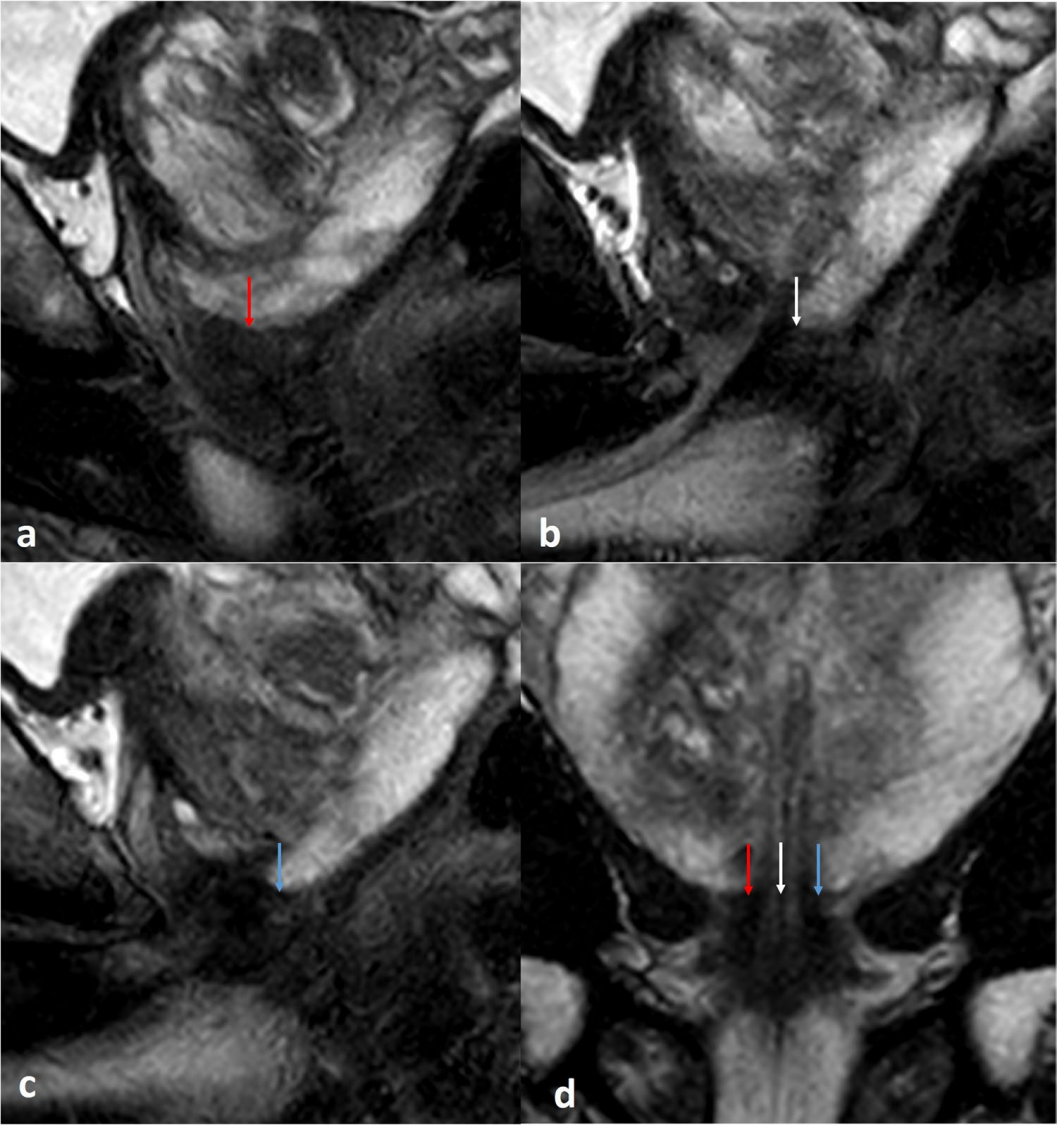
Challenges in membranous urethral length (MUL) measurement — the upper border, intersecting with the peripheral zone (1). Identifying the lower border of the peripheral zone may be challenging. Scrolling through the sagittal T2w images may help in determining the upper border of the MU, by better identifying the lower border of the peripheral zone. At the upper border of the membranous urethra, the retroprostatic part of the rectovesical space surrounding the peripheral zone in the prostate apex could be difficult to distinguish from the peripheral zone. These structures have similar signal intensities to the peripheral zone on T2-weighted images, especially when peripheral zone is less hyperintense on T2w images due to inflammation, fibrosis, blood products, or cancer. Scrolling 1 or 2 slices right (**a**; red arrow) and left (**c**; blue arrow) from the midsagittal view (**b**; white arrow) may improve the determination of the peripheral zone, and subsequently the upper border of the MU. On midsagittal images of the prostate, the dark tissue in the membranous urethra lumen may extend intraprostatic. This intraprostatic tissue will most likely be resected and should therefore be excluded from measurement. **d** Coronal T2 image shows the sagittal orientation of the lower borders of the peripheral zone on right parasagittal image (red arrow), midsagittal image (white arrow), and left parasagittal image (blue arrow)

**Fig. 4:**
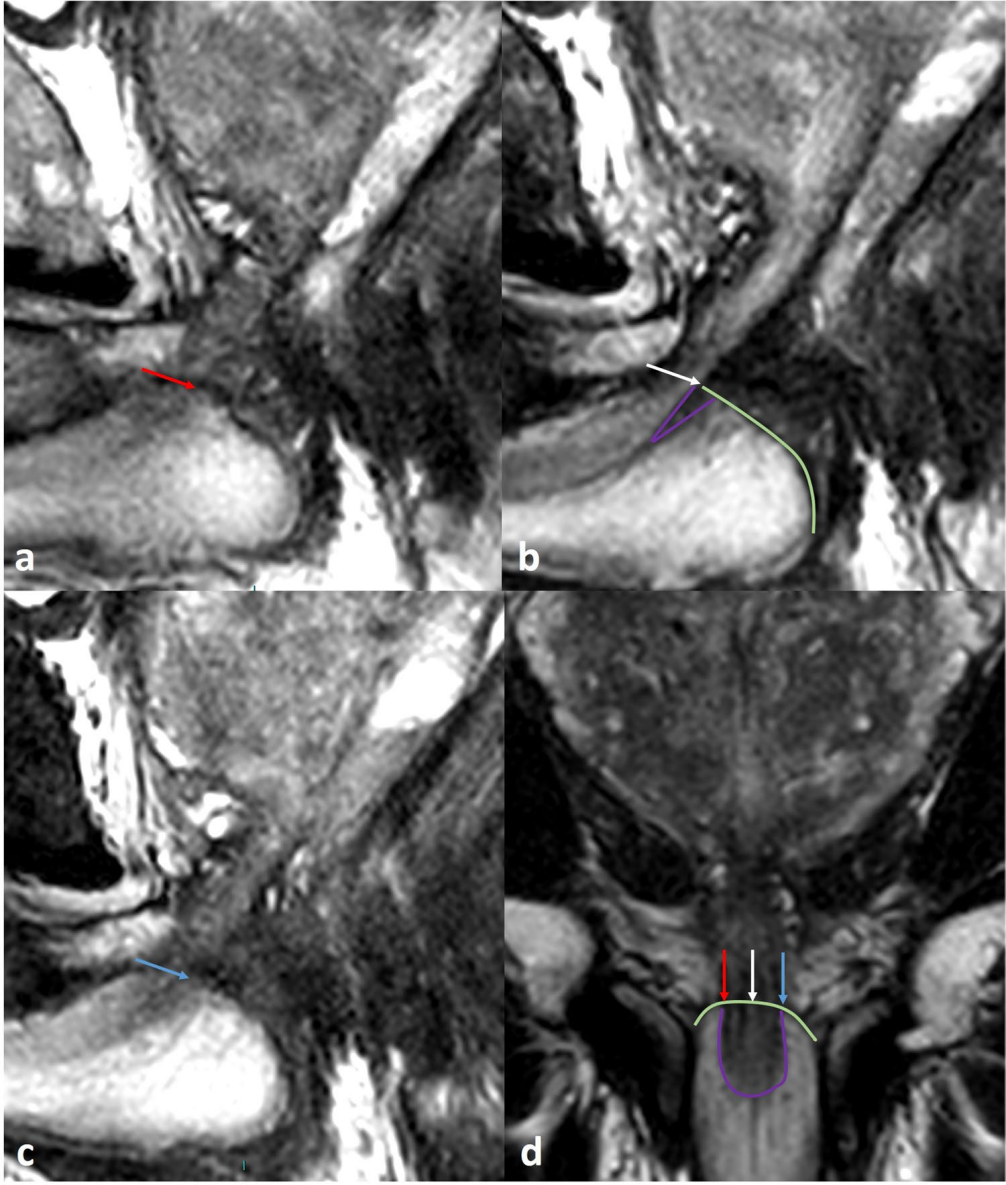
Challenges in membranous urethral length (MUL) measurement—the lower border, intersecting with the penile bulb (1). Scrolling through the sagittal T2w images may help in determining the lower border of the MU, by better identifying the upper border of the penile bulb. The lower border of the membranous urethra is determined by the intersection with the upper border of the penile bulb. Scrolling 1 or 2 slices right (**a**; red arrow) and left (**c**; blue arrow) from the midsagittal view (**b**; white arrow) may improve the determination of the penile bulb, and subsequently the lower border of the MU. On midsagittal images of the prostate, the hypointense tissue surrounding the membranous urethra lumen may continue into the penile bulb (purple in **b**). This intrabulbic part should be excluded from measuring. **d** Coronal T2 image shows the sagittal orientation of the upper borders of the penile bulb on right parasagittal image (red arrow), midsagittal image (white arrow), and left parasagittal image (blue arrow), and the intrabulbic continuation of hypointense tissue (purple line)

The proposed measurement and difference between coronal angulation and MUL measurement direction are shown in Fig. 1. The critical steps of our proposed MUL measurement technique are shown in Fig. 2. The identification of the upper limit (lower border of the peripheral zone) is illustrated by Fig. 3. The identification of the lower limit (upper border of the penile bulb) is illustrated by Fig. 4.

### Anatomy and measurement pitfalls

**Fig. 5:**
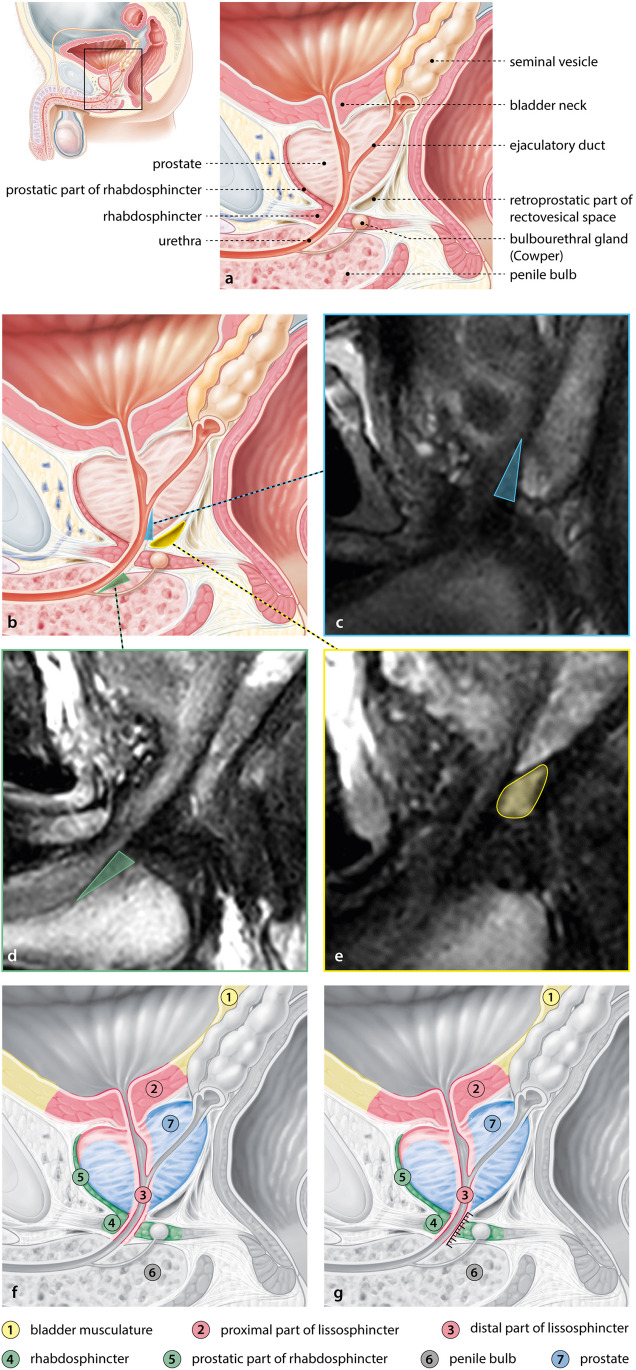
Complex anatomy of the region of the membranous urethra, correlation between MRI pitfalls, and the anatomy and the concept of the male urethral sphincter complex. The complex anatomy of the membranous urethra region in the midsagittal plane with the anatomical names (**a**). The areas on MRI that are responsible for the most important measurement pitfalls (**c**–**e**) with the corresponding area shown in the anatomical illustration (**b**). The pitfalls shown are fibres of the rhabdosphincter that appear to continue into the prostate (blue, **c**), dark tissue surrounding the urethra in the penile base (green, **d**), and the signal intensity of the retroprostatic part of the rectovesical space that can be similar to the peripheral zone (yellow, **e**). Concept of male urethral sphincter according to Koraitim with names (**f**, **g**). The MUL measurement with our proposed technique is indicated by the line (ruler) in **g**

The sphincter is composed of an external rhabdosphincter (skeletal muscle) that is responsible for the active continence and the internal lissosphincter (smooth muscle) that is responsible for the passive continence (Fig. 5f). The rhabdosphincter is the thickest at the level of the MU and has fibres continuous with the anterior fibromuscular stroma. The lissosphincter starts in the bladder neck and continues to the upper border of the penile base (perineal membrane). The MU is the part of the urethra between the prostatic apex and penile bulb (Fig. 5g). Both external and internal sphincter fibres are located at the level of the MU.

There are several pitfalls to consider when measuring the MU, resulting from the complex anatomy shown in Fig. 5a. In Fig. 5b–e, the correlation between the anatomy and the most important MRI pitfalls are shown. In Fig. 5f, g, the concept of the sphincter complex is shown according to Koraitim [13], showing a MUL measurement line in the anatomy image illustrating what is measured on MRI using our proposed technique (Fig. 5g).

**Fig. 6:**
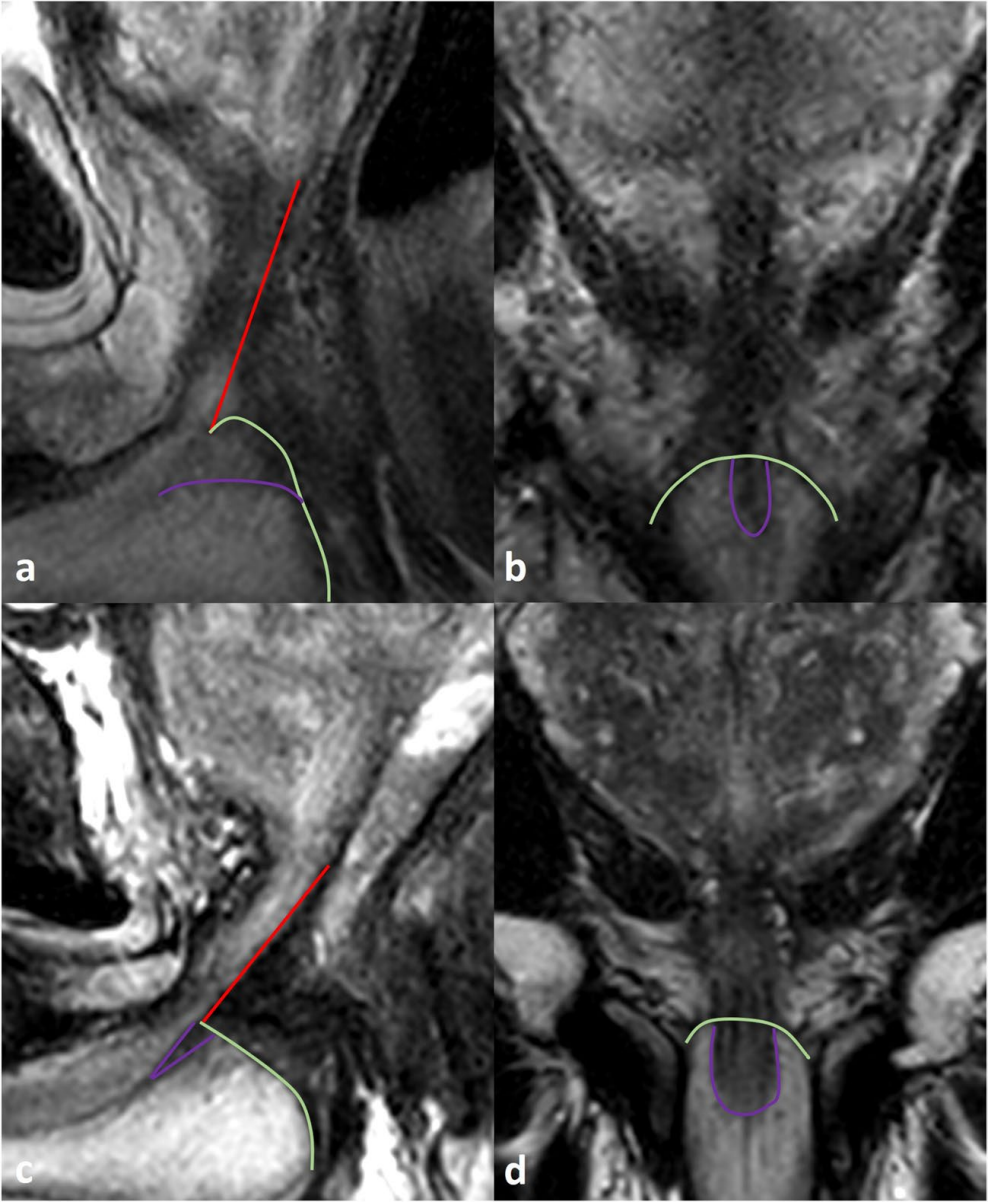
Pitfall in membranous urethral length (MUL) measurement—the lower border, intersecting with the penile bulb (2). The upper contour of the penile bulb may sometimes be difficult to determine at the midsagittal image. Peribulbic tissue surrounding the membranous urethra has various low T2 signal intensities. This tissue contains the perineal body, Cowper glands, and deep transverse perineal muscle. These structures are difficult to appreciate separately from the rhabdosphincter and similar dark signal intensity may continue several millimetres into the penile bulb. This may decrease the accurate demarcation of the lower border of the MU, resulting in extended MUL measurement with poor reproducibility. Scrolling 1 or 2 slices parasagittal (and when difficult) crosslinking with the coronal will help to identify the lower limit of the membranous urethra correctly. **a** A prostate cancer patient with sagittal T2w images on preoperative MRI. The proposed MUL measurement (red line) was challenging at the lower border, at the intersection with the penile bulb (green line). A ‘double contour’ appearance was suggested on the midsagittal image, as a result of partial volume effects (purple line). **b** Scrolling through the sagittal images left and right (not shown) and crosslinking with coronal images determined the appropriate upper contour of the penile bulb (green) and the intrabulbic hypointense tissue (purple line). **c** A prostate cancer patient with sagittal T2w images on preoperative MRI. The proposed MUL measurement (red line) was challenging at the lower border, at the intersection with the penile bulb (green line), due to the intrabulbic continuation hypointense tissue surrounding the urethra at the midsagittal image. **d** Scrolling through the sagittal images left and right (not shown) and crosslinking with coronal images determined the appropriate upper contour of the penile bulb (green) and the intrabulbic continuation of hypointense tissue surrounding the urethra (purple line)

Pitfalls include challenging superior limit (abnormal peripheral zone intensity, signal intensity of the retroprostatic part of the rectovesical space similar to the peripheral zone), challenging lower limit (double contour or difficulty to appreciate correct penile bulb contour at midsagittal slice (Fig. 6)), suggestion of rhabdosphincter fibers of MU continuing in the prostate (supplemental Fig. 1), angulated MU (supplemental Fig. 4), and crosslink errors between coronal and sagittal images (supplemental Fig. 5). It is important to have a good understanding of the anatomy of the MU and its surrounding structures. Additional text and illustrations on the anatomy and pitfalls are provided in the supplementary material, appendix 2.

## Discussion

The aim of the review was to investigate the MUL measurement and its interobserver agreement and propose literature-based recommendations to standardize MUL measurement for increasing interobserver agreement. To our knowledge, this is the first review to summarize the literature on MUL measurement methods and also the first to propose a standardized MRI-based MUL measurement approach with detailed landmarks and pitfalls. This could provide guidance for radiologists and urologists that would like to start performing these measurements as part of the preoperative risk assessment of postoperative urinary incontinence in men with localized prostate cancer. Standardization could also help to use externally validated urinary continence prediction tools.

### Populations

We observed that most literature on the MUL is from Asian countries. One study showed the average Asian MUL was significantly smaller than a non-Asian MUL [14]. The exact effect of different MUL size across populations (and whether this variation is associated with body length) should be further studied, as this may influence the continence prediction models suitable for different populations.

### Sequence and orientation

We observed that all studies that specified the image used for MUL measurement made use of T2-weighted images. Although most studies included sagittal images (sagittal only or both coronal and sagittal), a substantial number of publications used solely coronal images. The advantage of coronal images is that it allows easier delineation of upper and lower border. In literature, coronal and sagittal MUL measurements have shown significant correlation with urinary incontinence after RP and some studies showed good correlation between the sagittal and coronal measurements. We, however, recommend the use of sagittal images for MUL measurements. The angulation of coronal images is often different from the correct MU orientation that is seen in sagittal images. These variations in angulation will lead to different measurements compared with sagittal, causing under- or overestimation. Also, different coronal angulations will lead to different measurements in the same patient. Another theoretical possibility could be to angulate the coronal images parallel to the MU. However, it is questionable whether one should adjust the angulation and consequently the prostate appearance you are used to, especially for one measurement. Also, this requires training of radiologic technicians to accurately angulate parallel to the MU.

### Anatomical landmarks and line placement

We have seen that similar landmarks were used for upper and lower border of the measurement (‘prostatic apex’ and level/entry of the urethra at the penile bulb). However, the exact measurement descriptions in literature lacked details and are therefore poorly reproducible. For example, the exact measurement line location and orientation were not described and it was not mentioned how it was dealt with different apex types. All these factors can influence the MUL length. The transitional zone may be overlapping anteriorly [15] and it is unclear if measured towards prostate apex dorsally or anteriorly. The apical shape of the prostate is variable and may influence the predicted incontinence [16]. For reproducibility purposes, we suggest a standard measurement at the dorsal side of the MU towards the peripheral zone. To our knowledge, it is unknown whether measurement towards an apical protruding transitional zone is better for the predictive power of MUL measurements and intra- and interobserver agreement. In our experience, the dorsal side in easier to measure than central or anterior and parallel to the urethra would seem a rational approach.

### Measurement variations

The large variation in mean and median population MUL (median 7.3 to 17.3 mm) is suggesting large variation in measurement method or population. The large difference between these specific studies may be measurement method related, since both studies are from Japan.

### Observer agreement

Few studies reported on interobserver agreement variable results from fair to high agreement. In a recent agreement study from our group, we have seen high inter- and intraobserver agreement results using our defined landmarks [11]. It is important to obtain the highest possible intra- and interobserver agreement as a variation of several millimetres in MUL measurement results in substantially different percentage-predicted continence after RP.

### Imaging technique

We believe MRI is the technique of choice to use for the MUL measurements. It is possible to measure the MUL with other techniques, such as ultrasound and retrograde urethrography. However, the MRI is already made for targeting biopsy and/or staging and is able to visualize the anatomy very well.

### Our recommendations

For some of our measurement recommendations, there will be little discussion. The use of T2-weighted images is standard practice and the landmarks used are very similar in literature. Other recommendations may be a cause for more discussion. For example, the measurement on the midsagittal T2-weighted images, measuring dorsally along the urethra and towards the peripheral zone. Given the lack of evidence, we made these recommendations based on rationale and experience; this is a limitation.

### Other limitations

We did not study how interobserver agreement is of MUL measurements performed by readers (outside our institution) using our proposed measurement technique. Furthermore, the scanner type, coil type, and scan protocol may affect the image quality and appearance and therefore may influence the MUL measurement.

### Integration of MUL measurements in incontinence risk assessment following surgery

Because of the predictive power of the MUL, the authors believe that institutions are justified to implement the MUL measurements in clinical practise. The radiologists can provide the measurement in their standardized report, providing that the urologist knows how to interpretate the results and the radiologist is skilled in performing the measurement. Although the predictive power of the MUL has been proven in meta-analyses, the best way for the urologist to implement the measurement in decision-making can be debated. It is possible to use risk nomograms for personalized urinary incontinence risk to use for shared decision-making [3, 8]. Other methods could be to stratify patients into two or three categories of MUL size (e.g. high, intermediate, and low risk). Using these categories, small interobserver variations would lead to the same category. Although in some cases, a 1-mm difference may lead to a different risk category. At our own institution, we use the continence prediction tool (CPRED) which is based on the preoperative MRI-measured MUL, inner levator muscle distance (ILD), and the estimated extent of fascia preservation (i.e. nerve sparing) during RP [8]. However, the ILD is not as extensively studied as the MUL and the predictive power seems less compared with the MUL.

The recommended standardized MUL measurement needs to be validated and consensus among experts needs to be encouraged, including expert opinions.

## Conclusions

In order to improve measurement variability, a literature-based method for measuring the MUL was proposed, supported by several illustrative case studies, in an attempt to standardize MRI-based MUL measurements for appropriate urinary incontinence risk assessment following radical surgery.

### Supplementary information

Below is the link to the electronic supplementary material.

Supplementary file1 (PDF 526 KB)

## Abbreviations

BMI: Body mass index
Cor: Coronal
CPRED: Continence prediction tool
FSE: Fast spin echo
ICC: Intraclass correlation coefficient
LUTS: Lower urinary tract symptoms
MRI: Magnetic resonance imaging
MU: Membranous urethra
MUL: Membranous urethral length
NA: Not available
PI-RADS: Prostate Imaging – Reporting and Data System
PRISMA: Preferred Reporting Items for Systematic Reviews and Meta-analyses
RP: Radical prostatectomy
Sag: Sagittal
TSE: Turbo spin echo

## Declarations

### Guarantor

The scientific guarantor of this publication is Dr. Ivo Schoots.

### Statistics and biometry

No complex statistical methods were necessary for this paper.

### Methodology

• Review
