# Supplementary material for: Membranous urethral length measurement on preoperative MRI to predict incontinence after radical prostatectomy: a literature review towards a proposal for measurement standardization
Source: Eur Radiol. 2023 Sep 22;34(4):2621–40. doi: 10.1007/s00330-023-10180-7 (PMC10957670; doi:10.1007/s00330-023-10180-7)
Supplement: Supplementary file 1 — Supplementary file1 (PDF 526 KB) [file 330_2023_10180_MOESM1_ESM.pdf]

Membranous urethral length measurement on preoperative MRI to predict incontinence after radical prostatectomy: a literature review towards a proposal for measurement standardization

Electronic Supplementary Material

Appendix 1. Search strategy

MRI urinary continence prostate cancer

(Date of inception after each database)

Database searched	Records	Records after duplicates removed
Embase.com (1971-)	1350	1311
Medline ALL Ovid (1946-)	431	78
Web of Science Core Collection (1975-)	395	60
Cochrane CENTRAL register of trials (1992-)	11	23
Google scholar	100	45
**Total**	**2307**	**1505**

Embase.com (1971-)

('prostate tumor'/exp/dm_su OR 'prostate tumor'/exp/dm_rt OR ('prostate tumor'/exp AND ('focal therapy'/de OR 'cryoablation'/de OR 'cancer surgery'/de OR 'active surveillance'/de OR 'watchful waiting'/de OR 'cryotherapy'/exp OR radiotherapy/exp)) OR 'prostatectomy'/exp OR (((prostat*) NEAR/6 (cancer* OR carcinom* OR adenocarcinom* OR neoplas* OR tumo* OR lesion* OR oncolog*) NEAR/10 (surg* OR operat* OR postsurg* OR postoperat* OR radiotherap* OR irradiation* OR radiolog* OR focal-therap* OR cryotherap* OR cryosurg* OR cryoablat* OR cryo-ablat* OR Brachytherap* OR active-surveillan* OR watchful-wait*)) OR prostatectom* OR (prostat* NEAR/3 (resection OR adenectom*))):ab,ti) AND ('nuclear magnetic resonance imaging'/exp OR 'nuclear magnetic resonance'/exp OR (mri OR mpmri OR (MR NEAR/3 imag*) OR (magnetic* NEAR/3 resonan*)):ab,ti) AND ('urinary continence'/de OR 'urine incontinence'/exp OR incontinence/de OR continence/de OR 'functional outcome'/exp OR 'urine retention'/de OR 'International Prostate Symptom Score'/de OR 'micturition'/de OR 'micturition disorder'/de OR (continen* OR incontinen* OR retention OR (function* NEAR/3 outcome*) OR micturition OR urination):ab,ti) NOT ([conference abstract]/lim AND [1800-2017]/py) AND [English]/lim NOT ([animals]/lim NOT [humans]/lim)

Medline ALL Ovid (1946-)

(exp Prostatic Neoplasms/su OR exp Prostatic Neoplasms/rt OR (exp Prostatic Neoplasms/ AND (Cryosurgery/ OR Watchful Waiting/ OR Cryotherapy/ OR Radiotherapy/)) OR Prostatectomy/ OR (((prostat*) ADJ6 (cancer* OR carcinom* OR adenocarcinom* OR neoplas* OR tumo* OR lesion* OR oncolog*) ADJ10 (surg* OR operat* OR postsurg* OR postoperat* OR radiotherap* OR irradiation* OR radiolog* OR focal-therap* OR cryotherap* OR cryosurg* OR cryoablat* OR cryo-ablat* OR Brachytherap* OR active-surveillan* OR watchful-wait*)) OR prostatectom* OR (prostat* ADJ3 (resection OR adenectom*))).ab,ti.) AND (exp Magnetic Resonance Imaging/ OR exp Magnetic Resonance Spectroscopy/ OR (mri OR mpmri OR (MR ADJ3 imag*) OR (magnetic* ADJ3 resonan*)).ab,ti.) AND (exp Urinary Incontinence/ OR Urinary Retention/ OR Urination/ OR Urination

Disorders/ OR (continen* OR incontinen* OR retention OR (function* ADJ3 outcome*) OR micturition OR urination).ab,ti.) AND english.la. NOT (exp animals/ NOT humans/)

Web of Science Core Collection (1975-)

TS=(((((prostat*) NEAR/5 (cancer* OR carcinom* OR adenocarcinom* OR neoplas* OR tumo* OR lesion* OR oncolog*) NEAR/10 (surg* OR operat* OR postsurg* OR postoperat* OR radiotherap* OR irradiation* OR radiolog* OR focal-therap* OR cryotherap* OR cryosurg* OR cryoablat* OR cryo-ablat* OR Brachytherap* OR active-surveillan* OR watchful-wait*)) OR prostatectom* OR (prostat* NEAR/2 (resection OR adenectom*)))) AND ((mri OR mpmri OR (MR NEAR/2 imag*) OR (magnetic* NEAR/2 resonan*))) AND ((continen* OR incontinen* OR retention OR (function* NEAR/2 outcome*) OR micturition OR urination))) AND DT=(article) AND LA=(english)

Cochrane CENTRAL register of trials (1992-)

((((prostat*) NEAR/6 (cancer* OR carcinom* OR adenocarcinom* OR neoplas* OR tumo* OR lesion* OR oncolog*) NEAR/10 (surg* OR operat* OR postsurg* OR postoperat* OR radiotherap* OR irradiation* OR radiolog* OR focal-therap* OR cryotherap* OR cryosurg* OR cryoablat* OR cryo-ablat* OR Brachytherap* OR active-surveillan* OR watchful-wait*)) OR prostatectom* OR (prostat* NEAR/3 (resection OR adenectom*))):ab,ti) AND ((mri OR mpmri OR (MR NEAR/3 imag*) OR (magnetic* NEAR/3 resonan*)):ab,ti) AND ((continen* OR incontinen* OR retention OR (function* NEAR/3 outcome*) OR micturition OR urination):ab,ti)

Google scholar

"prostate|prostatic cancer|carcinoma|adenocarcinoma|neoplasms|tumors"
surgery|operative|radiotherapy|cryotherapy|cryoablation|prostatectomy mri|"magnetic resonance"
continence|incontinence|continent|incontinent

Appendix 2. Anatomy membranous urethra and surrounding structures

Prostate

The prostate is divided in three parts from cranial to caudal: the base, the midgland and the apex. The prostate consists of four histologic zones: the peripheral zone, the transitional zone, the anterior fibromuscular stroma and the central zone. The normal peripheral zone in adult men is homogenously hyperintense on the T2-weighted images. Although in practise many peripheral zones signal intensity is not that high, for example due to prostatitis, blood or tumour. The peripheral zone of the prostate is surrounded by a capsule. This 'capsule' is not considered to be a true capsule and is variable in conspicuity.

Urethra anatomy and function

The male urethra runs from the bladder neck to the external meatus and consists of four parts: the prostatic, membranous, bulbar and penile urethra. The prostatic urethra is surrounded by the transitional zone proximally and distally by the peripheral zone. The urethral sphincter consists of an inner and an outer sphincter. The inner (lisso) sphincter extends from the vesical orifice to the perineal membrane (cranial against the penile bulb), consists of cylindrical smooth muscle fibres around the urethra and is thickest around the bladder orifice. The external (rhabdo) sphincter is composed of skeletal muscle and is thickest around the membranous urethra (MU). Its fibres are continues with the anterior fibromuscular stroma. The inner sphincter is responsible for the passive incontinence (i.e., constant), while the external sphincter is responsible for the active continence (i.e., voluntary) [62]. After prostatectomy the only inner sphincter muscle fibres left for the passive continence are situated within the MU. The anatomy described above is illustrated in Figure 5 and based on the revised MU anatomy published by Koraitim [62]. The size of the MU is highly variable, from about 5 mm to over 35 mm (supplemental Figure 2).

Structures surrounding the membranous urethra

Levator ani muscle: On both sides of the MU a T2 hypointense levator ani muscle is seen (supplemental Figure 3). As these muscles could provide support to the MU, several parameters related to these muscles have been studied to predict incontinence (e.g., outer levator distance, inner levator distance, levator muscle thickness and perfusion quality of the levator muscle) [6].

The retroprostatic part of rectovesical space is located at the dorsal side just caudal from the peripheral zone of the prostate (supplemental Figure 3). The signal can be very close to the signal of the peripheral zone of the prostate.

Several structures are difficult to appreciate separately dorsal from the MU and caudally from the veins below the peripheral zone, these include: perineal body, Cowper glands, deep transverse perineal muscle. These structures can appear continuous with the externa sphincter muscles of the MU (supplemental Figure 3).

Penile base

The penis consists of corpus spongiosum located in the midline with the two corpora cavernosa alongside. All three corpora have similar hyperintense signal on T2 weighted images. The penile bulb is formed by the bulb of the corpus spongiosum.

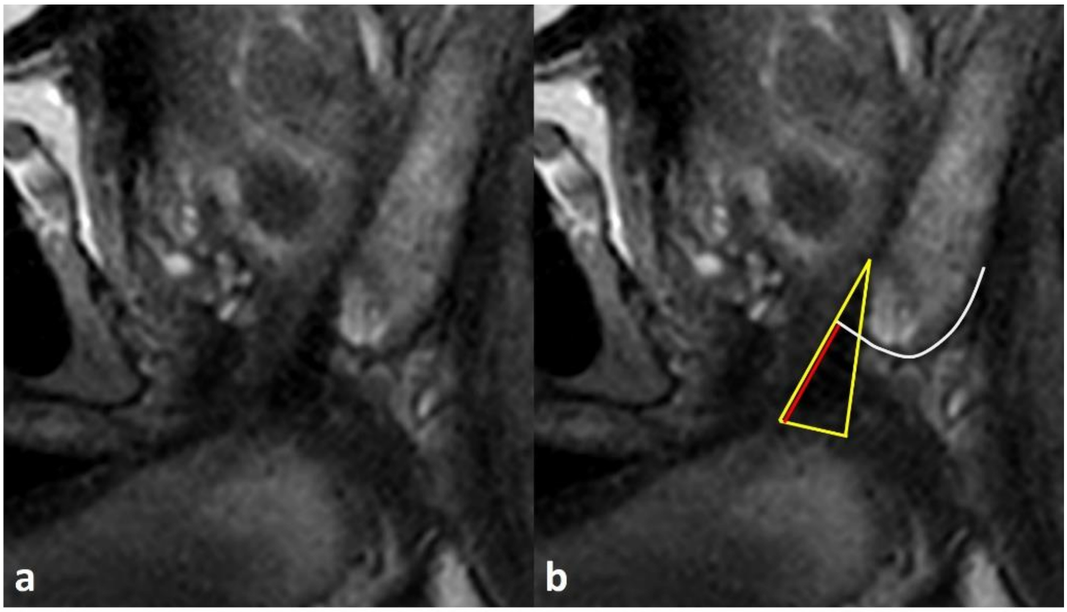

Supplemental Figure 1. Pitfall in membranous urethral length (MUL) measurement – the upper border, intersecting with the peripheral zone (2). The upper border of the MUL may sometimes be difficult to determine at the midsagittal image. a) a prostate cancer patient with sagittal T2w-images on pre-operative MRI. The proposed MUL measurement (red line) was challenging as a result of 'hypertropic' membranous urethra or rhabdosphincter, with intraprostatic continuation hypointense tissue that surrounds the lumen (yellow triangle) at the midsagittal image. b) the membranous urethra should be measured up to the lower border of the peripheral zone (white line), resulting in the proposed MUL measurement of 11 mm.

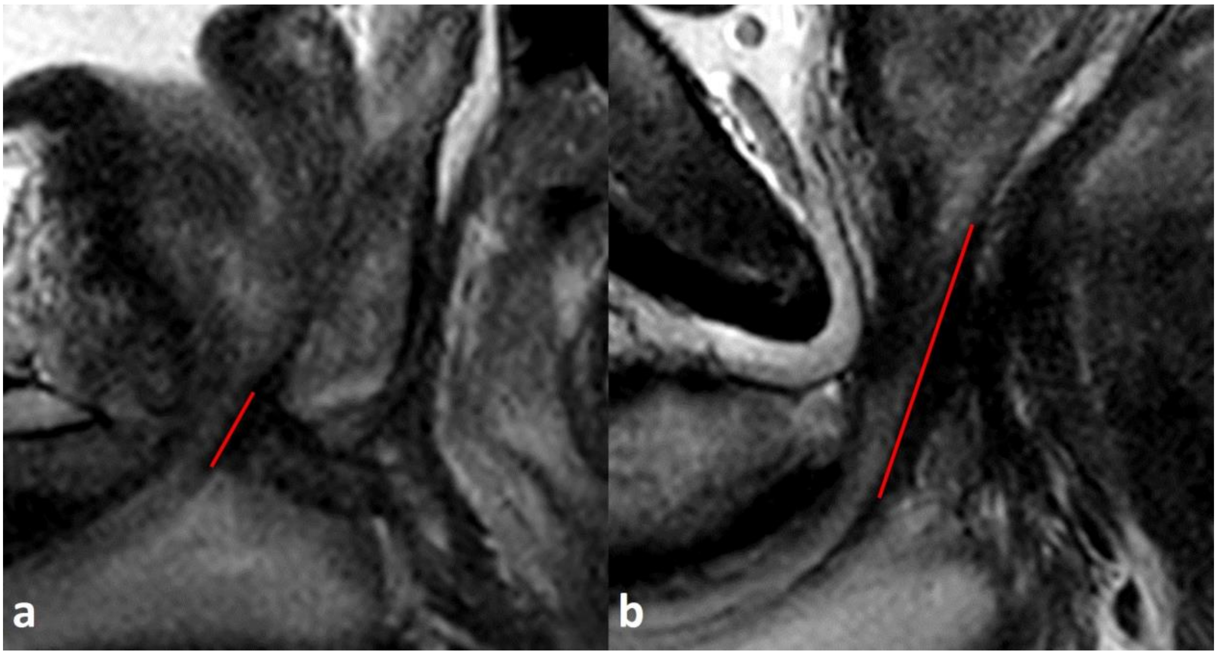

Supplemental Figure 2. Large variation in MUL a) Midsagittal T2w image of an 8 mm membranous urethral length (MUL) measurement (red line), which is short. b) Midsagittal T2w image of a 35 mm MUL (red line), which is long.

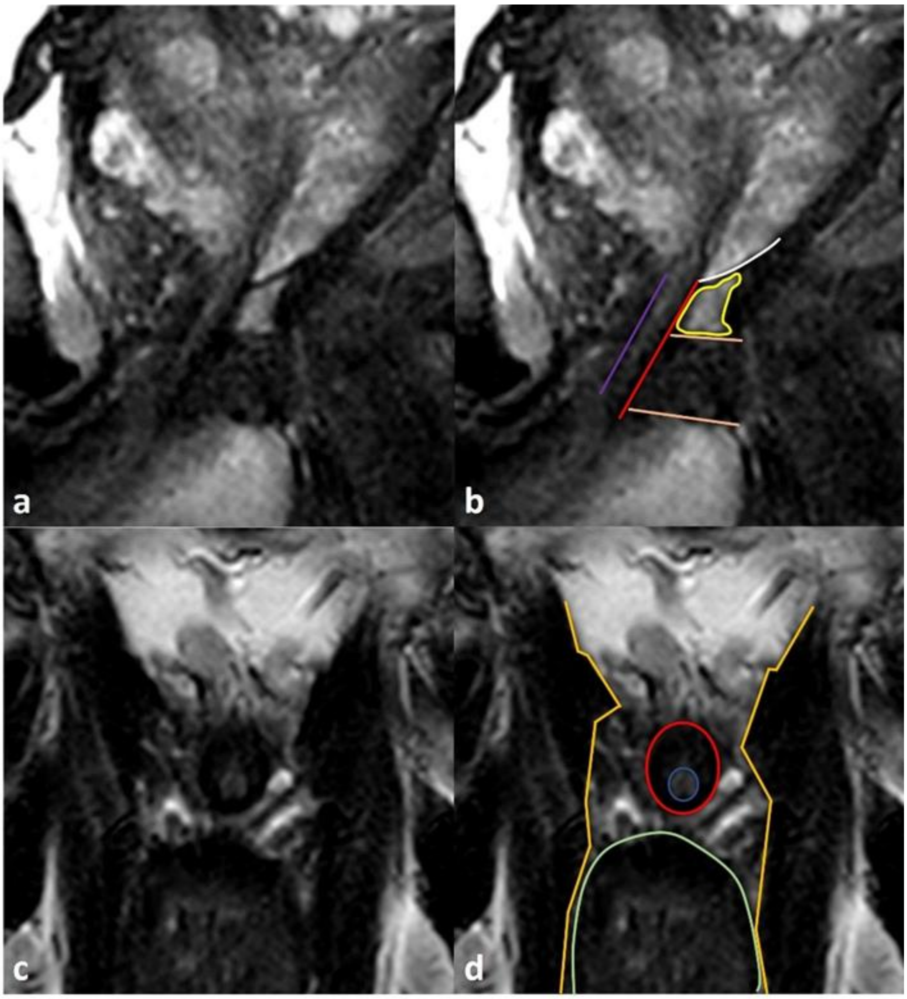

Supplemental Figure 3. MRI anatomy of the MU and surrounding structures. It is important to know the anatomy. For example, the venous plexus can have similar intensity to the peripheral zone. Sagittal (a and b) and axial T2 TSE images (c and d). a and c without and b and d with annotations. The lower border of the peripheral zone of the prostate is delineated with white. The hyperintense lumen of the MU can be seen between the purple and red line. The retroprostatic part of rectovesical space is located at the dorsal side just caudal from the peripheral zone of the prostate and is indicated with the yellow line. Several anatomical structures lie between the two pink line and include perineal body, Cowper glands, deep transverse perineal muscle. These structures are difficult to appreciate separately from the rhabdosphincter. In b the outer border of the rectal wall (green). Outer border of the rhabdosphincter (red). The levator ani muscle inner border (orange). Between these structures venous plexus can be seen. The MU lumen (blue).

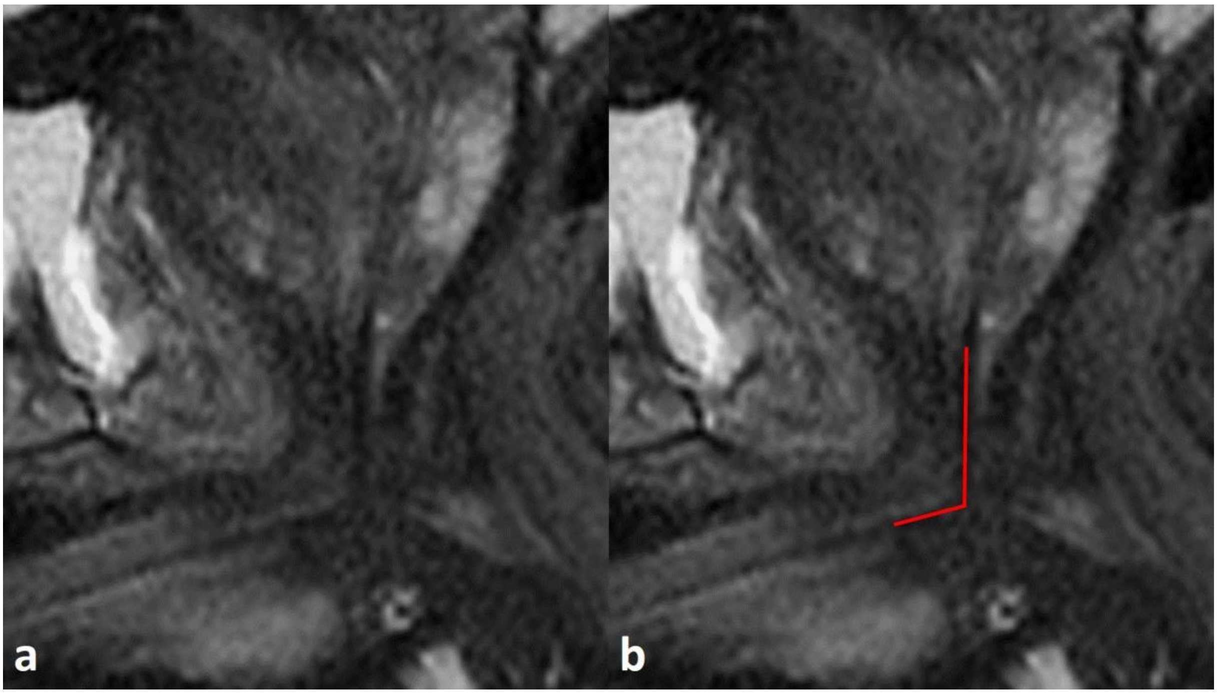

Supplemental Figure 4. Challenges in membranous urethral length (MUL) measurement – the lower border, intersecting with the penile bulb (2). a) the sagittal T2w-images on pre-operative MRI show an angulated membranous urethra. No specific literature was found on this topic, and per-operative continence predictions may be inappropriate on the MUL measurement attempt. b) An angulated measurement may at best approximate the MUL measurement (red line).

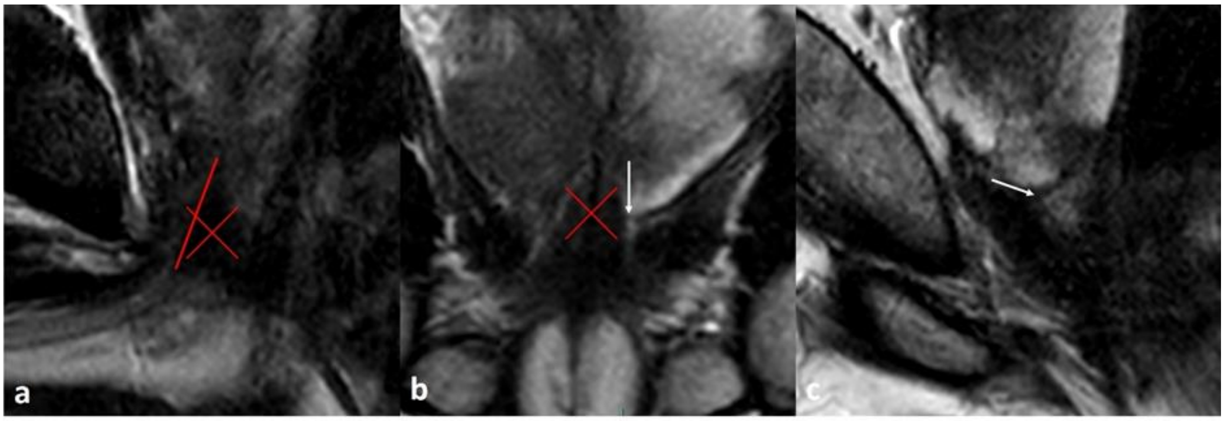

Supplemental Figure 5. Crosslink error. Although crosslinking with the coronal images often helps to determine correct borders, sometimes there is a mismatch due to patient movement between acquisitions. Therefore, critically assess whether crosslink seems reliable. Notice that the crosslink tool location in the midsagittal image (red cross in a) does not correspond with the red cross at the lower PZ border in the coronal image (b). In this case it is more helpful to scroll to parasagittal images (c). In this case left parasagittal images are better because there is tumour on the right with hypointense signal (b), making the capsule less clear.
